# Association of Wearable Device Use With Pulse Rate and Health Care Use in Adults With Atrial Fibrillation

**DOI:** 10.1001/jamanetworkopen.2021.5821

**Published:** 2021-05-27

**Authors:** Libo Wang, Kyron Nielsen, Joshua Goldberg, Jeremiah R. Brown, John S. Rumsfeld, Benjamin A. Steinberg, Yue Zhang, Michael E. Matheny, Rashmee U. Shah

**Affiliations:** 1 Division of Cardiovascular Medicine, University of Utah School of Medicine, Salt Lake City; 2 Department of Internal Medicine, University of Utah, Salt Lake City; 3 Department of Epidemiology, Geisel School of Medicine at Dartmouth, Hanover, New Hampshire; 4 Department of Biomedical Data Science, Geisel School of Medicine at Dartmouth, Hanover, New Hampshire; 5 University of Colorado School of Medicine, Aurora; 6 Study Design and Biostatistics Center, Center for Clinical and Translational Science, University of Utah, Salt Lake City; 7 Department of Biomedical Informatics, Vanderbilt University Medical Center, Nashville, Tennessee; 8 Department of Biostatistics, Vanderbilt University Medical Center, Nashville, Tennessee; 9 Department of Medicine, Vanderbilt University Medical Center, Nashville, Tennessee; 10 Geriatric Research Education and Clinical Center, Tennessee Valley Healthcare System, Nashville VA Medical Center, Nashville

## Abstract

**Importance:**

Increasingly, individuals with atrial fibrillation (AF) use wearable devices (hereafter *wearables*) that measure pulse rate and detect arrhythmia. The associations of wearables with health outcomes and health care use are unknown.

**Objective:**

To characterize patients with AF who use wearables and compare pulse rate and health care use between individuals who use wearables and those who do not.

**Design, Setting, and Participants:**

This retrospective, propensity-matched cohort study included 90 days of follow-up of patients in a tertiary care, academic health system. Included patients were adults with at least 1 AF-specific *International Statistical Classification of Diseases and Related Health Problems, Tenth Revision *(*ICD-10*) code from 2017 through 2019. Electronic medical records were reviewed to identify 125 individuals who used wearables and had adequate pulse-rate follow-up who were then matched using propensity scores 4 to 1 with 500 individuals who did not use wearables. Data were analyzed from June 2020 through February 2021.

**Exposure:**

Using commercially available wearables with pulse rate or rhythm evaluation capabilities.

**Main Outcomes and Measures:**

Mean pulse rates from measures taken in the clinic or hospital and a composite health care use score were recorded. The composite outcome included evaluation and management, ablation, cardioversion, telephone encounters, and number of rate or rhythm control medication orders.

**Results:**

Among 16 320 patients with AF included in the analysis, 348 patients used wearables and 15 972 individuals did not use wearables. Prior to matching, patients using wearables were younger (mean [SD] age, 64.0 [13.0] years vs 70.0 [13.8] years; *P* < .001) and healthier (mean [SD] CHA_2_DS_2_-VASc [congestive heart failure, hypertension, age ≥ 65 years or 65-74 years, diabetes, prior stroke/transient ischemic attack, vascular disease, sex] score, 3.6 [2.0] vs 4.4 [2.0]; *P* < .001) compared with individuals not using wearables, with similar gender distribution (148 [42.5%] women vs 6722 women [42.1%]; *P* = .91). After matching, mean pulse rate was similar between 125 patients using wearables and 500 patients not using wearables (75.01 [95% CI, 72.74-77.27] vs 75.79 [95% CI, 74.68-76.90] beats per minute [bpm]; *P* = .54), whereas mean composite use score was higher among individuals using wearables (3.55 [95% CI, 3.31-3.80] vs 3.27 [95% CI, 3.14-3.40]; *P* = .04). Among measures in the composite outcome, there was a significant difference in use of ablation, occurring in 22 individuals who used wearables (17.6%) vs 37 individuals who did not use wearables (7.4%) (*P* = .001). In the regression analyses, mean composite use score was 0.28 points (95% CI, 0.01 to 0.56 points) higher among individuals using wearables compared with those not using wearables and mean pulse was similar, with a −0.79 bpm (95% CI −3.28 to 1.71 bpm) difference between the groups.

**Conclusions and Relevance:**

This study found that follow-up health care use among individuals with AF was increased among those who used wearables compared with those with similar pulse rates who did not use wearables. Given the increasing use of wearables by patients with AF, prospective, randomized, long-term evaluation of the associations of wearable technology with health outcomes and health care use is needed.

## Key Points

**Question:**

Do individuals with known atrial fibrillation (AF) who use smartwatches or wearable devices use more health care resources and achieve better AF control?

**Findings:**

In this propensity-matched cohort study with 90-day follow-up among 16 320 patients with AF, individuals who used wearable devices had similar pulse rates compared with individuals who did not use these devices. Individuals using wearable devices had significantly increased rates of health care use; among use measures, the difference was significantly increased for ablation procedures.

**Meaning:**

These findings suggest that individuals with AF using wearable devices may have increased health care use but similar pulse rates on follow-up.

## Teaser

This cohort study investigates the association of wearable device use with pulse rate and health care use among adults with atrial fibrillation (AF).

## Introduction

Commercially available wearable devices (hereafter *wearables*) have flooded the market in recent years. Wearables are often marketed as health and wellness devices, with smartwatches and wristbands leading the wearable technology space. Hardware and software improvements have enabled additional features, such as heart rate monitoring, sleep monitoring, pulse oximetry, and respiration tracking. These advances open up new possibilities for disease-specific screening and management.^1,2,3^ Several devices cleared by the Food and Drug Administration (FDA) (eg, Apple Watch^4^ and AliveCor Kardia Band^5^) allow individuals to generate a single lead electrocardiogram (ECG) to screen for arrhythmias, such as atrial fibrillation (AF). Studies from 2019^6,7^ and 2020^8^ found that such devices were reasonably sensitive and specific in diagnosing new-onset AF, albeit among populations with high disease prevalence. The FDA recently reorganized the Center for Device and Radiological Health to reduce burdensome premarket approval processes. This is likely to increase the number of wearables available on the market.^9^

As a result of this market evolution, wearables are commercially available and commonly used by patients. While wearables measure pulse rate and, in some cases, alert individuals for incident AF events,^9^ the associations of these devices with health care use and clinical outcomes among patients with known AF are unknown. A looming clinical concern is that wearables may lead to an excessive number of false positives or true positive but nonactionable alerts, increasing health care use—or worse, increasing treatment-associated complications—without improvement in morbidity or mortality.^10,11,12^ And despite a lack of FDA clearance for the use of these devices among individuals with known arrhythmias, individuals with AF are using wearables with pulse rate and ECG capabilities. The goal of this exploratory project was to characterize individuals with AF who used and sought care based on a wearable’s output and to compare subsequent pulse rate and health care use between individuals who used wearables and those who did not. We hypothesized that wearable use among patients with AF would be associated with increased health care use.

## Methods

This cohort study was approved with waiver of informed consent granted by the institutional review board at the University of Utah because this was for retrospective research. This study is reported following the Strengthening the Reporting of Observational Studies in Epidemiology (STROBE) reporting guideline. The main objectives of our study were to compare the characteristics of individuals using wearables with those not using wearables and compare subsequent pulse rate and health care use patterns of individuals using wearables with those not using wearables.

### Patient Population and Exposures

**Figure 1.**
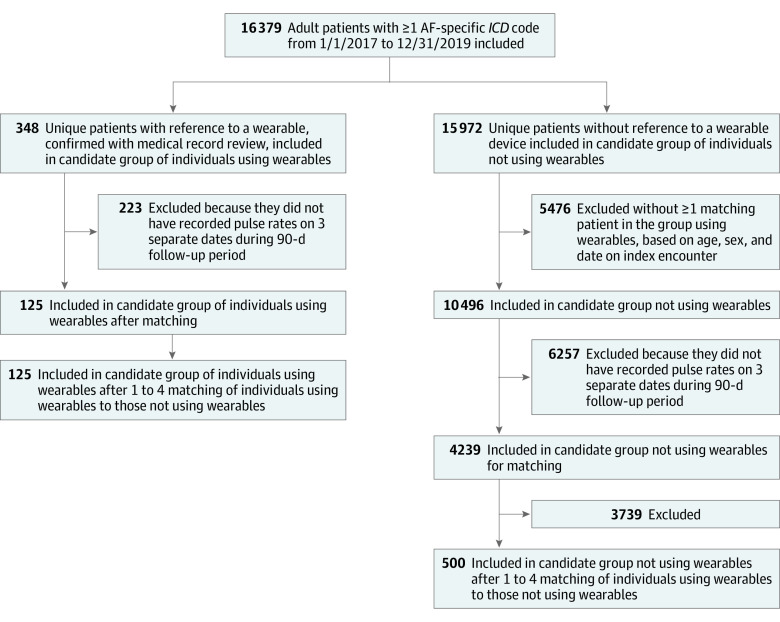
Study Population Selection Flowchart Study population selected from all patients with at least 1 *International Classification of Diseases *(*ICD*) code for atrial fibrillation (AF) from 2017 through 2019.

Data for this study were extracted from the enterprise data warehouse (EDW) at University of Utah Health. The EDW aggregates data from sources across the health system for use in operations and research efforts. Adult patients with at least 1 AF-specific *International Statistical Classification of Diseases and Related Health Problems, Tenth Revision *(*ICD-10*) code (ie, I48.0, I48.1, I48.11, I48.19, I48.2, I48.20, or I48.21) from January 1, 2017, through December 31, 2019, comprised the candidate population for these analyses. Figure 1 depicts the cohort assembly process. For these candidate patients, clinical notes were extracted as text files, and search terms specific to wearables were identified using regular expression matching (eg, “kardia”; eTable 1 in the Supplement). This approach yielded 786 notes for 452 unique patients with at least 1 term specific to a wearable. If a relevant term was present in a note, the text from that note was populated into a Research Electronic Data Capture form and manually reviewed to identify individuals using wearables based on the description and context. Patients with AF who did not have a note that included a term specific to a wearable were included in the group of individuals who did not use wearables. This population was used to compare general characteristics of individuals who did and did not use wearables (ie, the study’s first objective).

### Initial Exclusions Prior to Propensity Matching

We used a 2-stage process (ie, initial inclusion criteria followed by propensity matching) to control for confounding as rigorously as possible and to create the final cohort for comparison between individuals who did and did not use wearables (Figure 1). In the first step, initial criteria were applied to the nonusing candidate pool. Candidates who did not use wearables were included if they matched at least 1 individual who used wearables based on sex, year of birth (within 1 year), and index date, defined as follows. Candidates for the nonusing group had to have been seen for AF (defined by a documented relevant *ICD* code) within 60 days of at least 1 of the encounters by a member of the using group that referred to a wearable. Each candidate from the 2 groups was assigned an index date that served as day 0 in the analyses. For individuals using wearables, the index date was defined as the earliest date of the note that included a reference to a wearable within the study time frame. For individuals not using wearables, the index date was defined as the date of an encounter that included an AF-specific billing code and occurred within 60 days of an index date for an individual using wearables. If multiple dates were available, the earliest date during the 60-day interval was used.

For both groups, we excluded patients whose index date occurred after September 30, 2019, and those with insufficient follow-up data during the 90-day outcome time period (ie, those with fewer than 3 unique dates with a recorded pulse rate). This approach allowed for a more rigorous comparison in this observational analysis because individuals who did and did not use wearables were seen at about the same time and all included patients had sufficient follow-up data.

### Propensity Matching After Initial Exclusions

Individuals who did not use wearables were then matched to those who used wearables using propensity score matching at a ratio of 4 to 1.^13^ Specifically, we used a logistic regression model to estimate the propensity score, defined as the probability of being an individual who used wearables, as a function of variables that were potentially associated with choice of using a wearable for each individual in the cohort. Socioeconomic status is an important confounder in studies examining consumer technologies. We accounted for socioeconomic status by using the area deprivation index of the patient’s zip code within 90 days of the index date, with a higher area deprivation index indicating increased deprivation.^14^ The variables in the propensity score model included patient characteristics present at the time of and prior to the index date: age, sex, area deprivation index, index year, first visit for AF, number of days between index date and first AF visit in our system, baseline pulse, baseline pulse ≥ 110 beats per minute (bpm), history of ablation, history of cardioversion, CHA_2_DS_2_-VASc (congestive heart failure, hypertension, age ≥ 75 or 65-74 years, diabetes, prior stroke/transient ischemic attack, vascular disease, sex) score ≥ 2, Charlson Comorbidity Index score,^15^ mean pulse value from prior visits, oral anticoagulant use, rate control drug use, rhythm control drug use, number of prior evaluation and management (EM) codes, and number of prior telephone notes. The matching was performed without replacement based on the distance between each observation of an individual who used wearables and the observations in its neighborhood of individuals who did not use wearables, using the propensity score as a metric of distance. Standardized mean differences were used to assess covariate balance after matching (eTable 2 in the Supplement).^16^

### Outcomes

The co-primary outcomes of interest were pulse rate and a composite health care use score, assessed over a 90-day period following the index date through December 31, 2019, to maintain a consistent follow-up time for all patients. Pulse rate was chosen as a surrogate for clinical outcome given that it is measured at almost all clinic visits and has prognostic value in AF.^17^ If multiple pulse rates were recorded on the same day for the same patient, the mean value for that day was used to avoid overcounting clinically active days (ie, procedures). Individuals who used wearables and those who did not use wearables were compared using the mean and median pulse rates from all outpatient-recorded pulse rates during the outcome period for each patient. Mean and median pulse rates were further divided into 5-unit intervals to approximate clinically relevant changes.

The composite use score was designed to approximate health care use specific to AF management. Prior methods to estimate use have focused on inpatient hospital days or, broadly, outpatient claims for AF.^18^ We chose a more specific approach, incorporating health care actions that could be triggered by information from a wearable (eg, cardioversions and prescription orders). Similar to prior approaches, we accounted for professional services (ie, EM codes), but we did not account for inpatient hospitalizations.^19^ An ordinal variable was created from components of clinical encounters and AF-specific treatments, ranging from 0 to 8, with higher values indicating more use. We found that 38 out of 575 patients (6.6%) had scores of 6, 7, or 8, so these values were aggregated into a single value of 6. The components of the use outcome were: (1) Number of encounters with an EM or ablation *Common Procedural Terminology* (*CPT*) code. This number was categorized into 3 groups: 0 if no codes were present, 1 if the number of codes present was below the median of the group, and 2 if the number of codes present was above the median of the group. (2) Number of encounters with a cardioversion *CPT* code. This number was categorized as 0 if no codes were present and 1 if at least 1 code was present. (3) Number of telephone encounter notes (ie, 0 if no notes, 1 if the number of notes was below the median, and 2 if the number of notes was above the median). (4) Number of rate control medication orders (ie, 0 if no orders and 1 if at least 1 order). (5) Number of rhythm control medication orders (ie, 0 if no orders and 1 if at least 1 order).

### Patient Characteristics

Prior conditions were identified from the presence of *ICD* billing codes prior to the index date. The EDW maintains an *ICD* code mapping to generate the Charlson Comorbidity Index score for each patient, using all diagnostic codes available in each patient's electronic medical record. We used *CPT* codes to identify EM visits (ie, codes 99201, 99202, 99203, 99204, 99205, 99212, 99213, 99214, 99215, 99281, 99282, 99283, 99284, 99285, 99304, 99305, 99306, 99307, 99308, 99309, 99310, 99324, 99325, 99326, 99327, 99328, 99334, 99335, 99336, 99337, 99341, 99342, 99343, 99344, 99345, 99347, 99348, 99349, and 99350), cardioversions (ie, code, 92960), and ablations (ie, codes, 93650, 93651, 93653, 93656, and 93657).

Similarly, the EDW maintains an autocalculated CHA_2_DS_2_-VASc score for AF patients using demographic characteristics and historical *ICD* diagnoses billing codes. Each time an AF-specific *ICD* code appears in the electronic medical record, the system generates a CHA_2_DS_2_-VASc score for the corresponding date. We used the score closest in time to the index date as the baseline score for these analyses.

### Statistical Analysis

Data cleaning, matching, and statistical analysis were performed using Python programming language version 3.8 (Python Software Foundation) and R statistical software version 3.6.0 (R Project for Statistical Computing). Characteristics of individuals who used and did not use wearables were compared using univariate logistic regression to generate odds ratios for binary variables and univariate linear regression to generate mean differences for continuous variables. Mixed-effects linear regression models were used to estimate the association between wearable use status and mean pulse rate, median pulse rate, and composite use score. The random component in the model was used to account for clustering structure associated with the matching procedure. *P* values were 2-sided, and statistical significance was set at .05. Data were analyzed from June 2020 through February 2021.

## Results

### Baseline Characteristics

Among 16 320 patients identified with at least 1 AF-specific *ICD* diagnosis billing code from January 1, 2017, through December 31, 2019, 452 patients had at least 1 note with a reference to a wearable, with 851 device mentions among 786 notes; the study population consisted of 348 patients who were determined to have used wearables among patients with a wearable mention and 15 972 patients with at least 1 AF-specific *ICD* code and no device mentions. Apple watch was the most frequently mentioned device, with 240 mentions, followed by Fitbit, with 200 mentions, and Kardia, with 153 mentions (eFigure in the Supplement). Baseline characteristics for individuals using wearables compared with individuals not using wearables, prior to any exclusions or matching, are found in Table 1. The individuals who used wearables, compared with individuals who did not use wearables, were younger (mean [SD] age, 64.0 [13.0] years vs 70.0 [13.8] years; *P* < .001), were healthier (mean [SD] CHA_2_DS_2_-VASc score, 3.6 [2.0] vs 4.4 [2.0]; *P* < .001), had similar gender proportion (148 [42.5%] women vs 6722 women [42.1%]; *P* = .91), were less socioeconomically deprived (mean [SD] area deprivation index, 28.1 [18.0] vs 37.7 [19.2]; *P* < .001), and had a lower mean (SD) Charlson Comorbidity Index score (3.3 [3.1] vs 4.0 [3.2]; *P* < .001). Individuals who used wearables had greater mean (SD) EM encounters (7.2 [7.6] vs 4.4 [6.2]; *P* < .001) and median (interquartile range [IQR]) number of telephone notes (13.0 [3.0-39.0] vs 1.0 [0-7.0]; *P* < .001) prior to the index date compared with individuals who did not use wearables.

**Table 1.** Patient Characteristics Prior to Exclusions and Matching

Characteristic	No. (%)		*P* value
	Candidates not using wearables (n = 15 972)	Candidates using wearables (n = 348)	
Age, mean (SD), y	70.0 (13.8)	64.0 (13.0)	<.001
Sex			
Women	6722 (42.1)	148 (42.5)	.91
Men	9250 (57.9)	200 (57.5)	
White race/ethnicity	14 214 (89.0)	328 (94.3)	.002
Area deprivation index, mean (SD)	37.7 (19.2)	28.1 (18.0)	<.001
No. prior EM *CPT* codes, mean (SD)	4.4 (6.2)	7.2 (7.6)	<.001
No. prior emergency department visits, mean (SD)	0.3 (1.4)	0.5 (1.4)	.01
≥1 prior AF ablation *CPT* code	261 (1.6)	34 (9.8)	<.001
≥1 prior cardioversion *CPT* code	363 (2.3)	53 (15.2)	<.001
≥1 prior anti-arrhythmic drug order	2411 (15.1)	114 (32.8)	<.001
≥1 prior rate control drug order	7673 (48.0)	222 (63.8)	<.001
Prior oral anticoagulant^a^	6404 (40.1)	220 (63.2)	<.001
No. prior ECGs, median (IQR)	0 (0-3.0)	4.0 (1.0-9.0)	<.001
No. dates with ≥ 1 pulse rate measurement, mean (SD)	41.6 (60.2)	56.8 (62.8)	<.001
Pulse rate at index visit, mean (SD)	76.8 (16.7)	72.9 (17.0)	<.001
Pulse rate at index visit >109 bpm	475 (3.0)	8 (2.3)	.57
Pulse rate prior to index date, mean (SD)	75.2 (11.2)	74.0 (11.5)	.05
CHA_2_DS_2_-VASc score			
0-2	2038 (12.8)	77 (22.1)	<.001
3-5	5466 (34.2)	126 (36.2)	
6+	3463 (21.7)	44 (12.6)	
CHA_2_DS_2_-VASc score >1	10 048 (91.6)	210 (85.0)	<.001
CHA_2_DS_2_-VASc score, mean (SD)	4.4 (2.0)	3.6 (2.0)	<.001
Charlson Comorbidity Index score, mean (SD)	4.0 (3.2)	3.3 (3.1)	<.001
No. prior telephone notes, median (IQR)	1.0 (0-7.0)	13.0 (3.0-39.0)	<.001
≥1 prior telephone note	8438 (52.8)	302 (86.8)	<.001
Time since first AF diagnosis, mean (SD), d	481.0 (823.4)	755.8 (882.3)	<.001
First AF visit	10 185 (63.8)	40 (11.5)	<.001

Abbreviations: AF, atrial fibrillation; bpm, beats per minute; CHA_2_DS_2_-VASc, congestive heart failure, hypertension, age 75 years or older, diabetes, prior stroke/transient ischemic attack–vascular disease, age 65 to 74 years, sex category; *CPT*, *Common Procedural Terminology*; EM, evaluation and management; ECG, electrocardiogram; IQR, interquartile range.

^a^ Oral anticoagulant order or appearance in listed medications as per the electronic medical record.

After initial exclusions and propensity matching, 125 individuals who used wearables and 500 individuals who did not use wearables were compared using the index date for baseline characteristics (Table 2). Age, sex, rhythm control strategies, comorbidities, CHA_2_DS_2_-VASc score, number of ECGs, and health care use were statistically similar in the matched group. Individuals who used wearables had more median (IQR) electrocardiograms prior to the index date compared with those who did not use wearables (5 [2-11] vs 4 [1-9]; *P* = .02). Mean (SD) pulse prior to the index date was similar among those who did and did not use wearables (75.3 [11.5] bpm vs 76.2 [11.0] bpm; *P* = .42), as was mean (SD) pulse at the time of the index (75.3 [18.0] bpm vs 76.5 [15.2] bpm, *P* = .51).

**Table 2.** Patient Characteristics After Exclusions and Propensity Matching

Characteristic	No. (%)		*P* value
	Not using wearables (n = 500)	Using wearables(n = 125)	
Age, mean (SD), y	64.6 (12.6)	63.1 (13.7)	.28
Sex			
Women	188 (37.6)	48 (38.4)	.95
Men	312 (62.4)	77 (61.6)	
White race/ethnicity	452 (90.4)	118 (94.4)	.22
Area deprivation index, mean (SD)	28.0 (16.7)	27.4 (18.6)	.73
No. prior EM *CPT* codes, mean (SD)	9.6 (8.1)	10.1 (10.0)	.62
No. prior emergency department visits, mean (SD)	0.7 (1.4)	0.8 (2.1)	.45
≥1 prior ablation *CPT* code	56 (11.2)	15 (12.0)	.93
≥1 prior cardioversion *CPT* code	90 (18.0)	26 (20.8)	.55
≥1 prior anti-arrhythmic drug order	185 (37.0)	44 (35.2)	.79
≥1 prior rate control drug order	344 (68.8)	85 (68.0)	.95
Prior oral anticoagulant^a^	376 (75.2)	94 (75.2)	>.99
No. prior ECGs, median (IQR)	4.0 (1.0-9.0)	5.0 (2.0-11.0)	.02
Pulse rate at index visit, mean (SD)	76.5 (15.2)	75.3 (18.0)	.51
Pulse rate prior to index visit, mean (SD)	76.2 (11.0)	75.3 (11.5)	.42
CHA_2_DS_2_-VASc score grouped			
0-2	96 (19.2)	25 (20.0)	.99
3-5	233 (46.6)	59 (47.2)	
6+	97 (19.4)	24 (19.2)	
CHA_2_DS_2_-VASc score >1	389 (91.3)	98 (90.7)	>.99
CHA_2_DS_2_-VASc score, mean (SD)	4.1 (1.9)	4.0 (1.8)	.64
Charlson Comorbidity Index score, mean (SD)	4.2 (3.2)	4.1 (3.2)	.78
No. prior telephone notes, median (IQR)	18.0 (5.0-42.0)	19.0 (4.0-59.0)	.41
≥1 prior telephone note	447 (89.4)	111 (88.8)	.98
Time since first AF diagnosis, mean (SD), d	764.0 (814.0)	782.8 (887.8)	.83
First AF visit	24 (4.8)	7 (5.6)	.89

Abbreviations: AF, atrial fibrillation; CHA_2_DS_2_-VASc, congestive heart failure, hypertension, age 75 years or older, diabetes, prior stroke/transient ischemic attack–vascular disease, age 65 to 74 years, sex category; *CPT*, *Common Procedural Terminology*; ECG, electrocardiogram; EM, evaluation and management; IQR, interquartile range.

^a^ Oral anticoagulant order or appearance in listed medications as per the electronic medical record.

### Follow-up Outcomes

**Figure 2.**
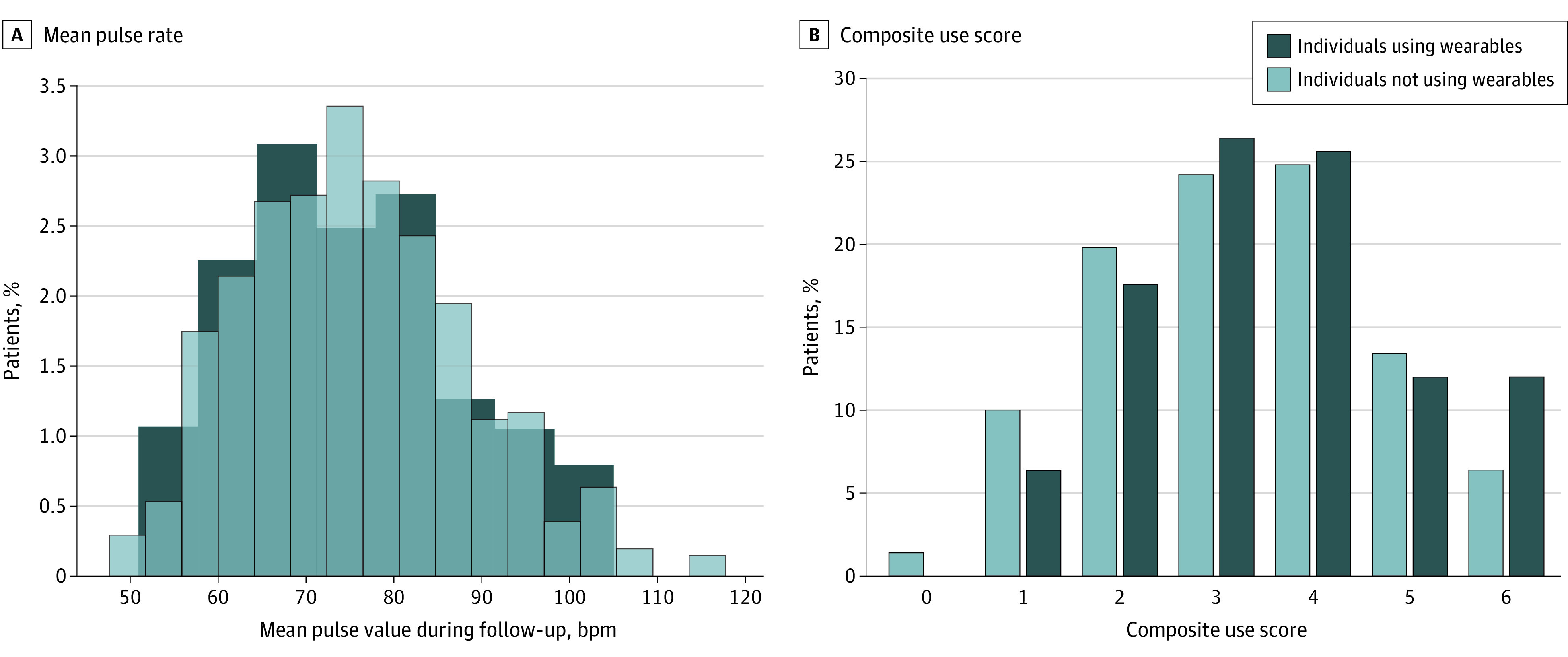
Mean Pulse Rate and Composite Health Care Use During Follow-up Period A, For each patient, mean value of all pulse rates in the 90-day period following the index date was calculated. B, Composite use score included evaluation and management encounters, cardioversions, telephone encounters, and rate and rhythm control medications. bpm indicates beats per minute.

During the follow-up period, the mean pulse rate was similar between the 2 groups (75.01 bpm [95% CI, 72.74-77.27] vs 75.79 [95% CI, 74.68-76.90] bpm; *P* = .54) (Table 3, Figure 2A), as was the mean of all median pulse rates (73.68 [95% CI, 71.44-75.92] bpm vs 75.03 [95% CI, 73.86-76.20] bpm, *P* = .30) (Table 3; eTable 3 in the Supplement). Overall use was higher during the follow-up period for individuals who used wearables (Figure 2B), with increased mean (3.55 [95% CI, 3.31-3.80] vs 3.27 [95% CI, 3.14-3.40]; *P* = .04) composite use scores, while the median (IQR) composite use score was similar for the 2 groups (3.0 [3.0-4.0] vs 3.0 [2.0-4.0]; *P* = .08). Among the measures in the composite outcome, there was a significant difference in use of ablation, with at least 1 ablation *CPT* code found in 22 individuals who used wearables (17.6%) vs 37 individuals who did not use wearables (7.4%) (*P* < .001). The number of telephone notes, rate control and anti-arrhythmic drug prescriptions, and cardioversions were similar between the 2 groups (Table 3).

**Table 3.** Outcomes of Matched Patients

Outcome	Not using wearables (n = 500)	Using wearables (n = 125)	Comparison
**Continuous outcome**	**Mean (95% CI)**	**Mean (95% CI)**	**Mean difference (95% CI)**
Composite use score	3.27 (3.14 to 3.40)	3.55 (3.31 to 3.80)	0.28 (0.01 to 0.56)
Pulse rate, mean values	75.79 (74.68 to 76.90)	75.01 (72.74 to 77.27)	−0.79 (−3.28 to 1.71)
Pulse rate, median values	75.03 (73.86 to 76.20)	73.68 (71.44 to 75.92)	−1.35 (−3.95 to 1.24)
No. anti-arrhythmic drug orders	0.11 (0.08 to 0.15)	0.17 (0.07 to 0.26)	0.05 (−0.03 to 0.14)
No. rate control drug orders	1.15 (0.96 to 1.35)	1.25 (0.86 to 1.63)	0.09 (−0.34 to 0.52)
No. telephone notes	14.83 (13.19 to 16.48)	15.09 (10.72 to 19.46)	0.25 (−3.70 to 4.21)
No. EM *CPT* codes	3.46 (3.21 to 3.71)	3.61 (3.13 to 4.08)	0.15 (−0.41 to 0.71)
No. cardioversion *CPT* codes	0.19 (0.13 to 0.24)	0.30 (0.15 to 0.44)	0.11 (−0.026 to 0.246)
No. ablation *CPT* codes	0.19 (0.13 to 0.26)	0.44 (0.26 to 0.62)	0.25 (0.09 to 0.41)
No. dates with ≥1 pulse rate measurement	14.81 (13.23 to 16.38)	17.11 (14.04 to 20.19)	2.31 (−1.19 to 5.81)
**Categorical outcome**	**Proportion, % (95% CI)**	**Proportion, % (95% CI)**	**OR (95% CI)**
≥1 Anti-arrhythmic drug order	9.2 (6.7 to 11.7)	12.0 (6.2 to 17.7)	1.36 (0.72 to 2.50)
≥1 Rate control drug order	36.6 (32.4 to 40.8)	40.0 (31.3 to 48.7)	1.15 (0.77 to 1.73)
≥1 Cardioversion *CPT* code	8.4 (6.0 to 10.8)	14.4 (8.2 to 20.6)	1.83 (1.02 to 3.31)
≥1 Ablation *CPT* code	7.4 (5.1 to 9.7)	17.6 (10.9 to 24.3)	2.67 (1.51 to 4.72)
Proportion with No. of EM codes above median	54.4 (50.0 to 58.8)	64.8 (56.4 to 73.2)	1.54 (1.03 to 2.32)
Proportion with No. of telephone notes above median	43.0 (38.7 to 47.3)	40.8 (32.1 to 49.5)	0.91 (0.61 to 1.36)

Abbreviations: *CPT*, *Common Procedural Terminology*; EM, evaluation and management.

In the regression analyses, which clusters based on propensity matching, the mean composite use score was 0.28 points (95% CI, 0.01 to 0.56 points) higher among individuals using wearables compared with those not using wearables. The mean pulse was similar, with a −0.79 bpm (95% CI, −3.28 to 1.71 bpm) difference, as was the mean of all median pulse rates, with a −1.35 bpm (95% CI, −3.95 to 1.24 bpm) difference. Because 1-unit differences in pulse may not be clinically meaningful, we also evaluated for 5-unit differences in pulse between the 2 groups and found no significant differences (eTable 3, eTable 4, and eTable 5 in the Supplement).

## Discussion

Wearable sales are booming.^20,21^ Large randomized clinical trials published in 2020^22,23^ focus on using such devices as screening tools among patients without arrhythmias. Our cohort study is among the first studies to systematically characterize the use of wearable use among individuals with AF as a part of routine health care delivery. Individuals who used wearables were younger, healthier, and socioeconomically better off than those who did not use wearables. We found no difference in clinic-measured pulse rates between the 2 groups but higher health care use rates among individuals who used wearables.

Like our results, those of prior studies raised questions about effective ways to apply wearable technology to improve health outcomes. A 2020 retrospective analysis^24^ of Apple Watch alerts found that 11% of alerts were associated with a clinically actionable event. Another study of inpatients on telemetry^7^ found poor sensitivity of smartwatch AF detection among patients after cardiac surgical treatment, a group known to have a high arrhythmia burden. These findings, along with those reported in our study, support the need for more disease-specific data to guide the clinical use of wearables.

Our observational analyses provide important information, given the absence of randomized trials in this population thus far. First, clinicians may be concerned about an excessive number of phone calls based on readings from a wearable; we found no evidence to suggest that pattern. Second, our data suggest that individuals who use wearables may be healthier than those who do not use wearables. This finding is unsurprising considering adoption patterns of new technology by younger patients, and it raises the possibility that subsequent clinical interventions based on wearables may not make patients feel better if they are already well. Third, most individuals in our study who used wearables had previously diagnosed AF. The wearable was not used for screening or diagnosis of AF, but rather for self-management; however, most of the published evidence focuses on these wearables as screening tools. These findings may have important implications for ongoing implementation of wearables into clinical care and their potential (or lack thereof) to improve personal health and health care delivery. Prospective, randomized clinical trials focused on wearable use among patients with AF are needed to fully understand the association of wearables with health outcomes.

Wearables have potential use in longitudinal care of the arrhythmic population and will likely play increasing roles in clinical management.^25^ Indeed, the coronavirus disease 2019 pandemic has accelerated the shift in health care delivery away from traditional settings, with increasing use of telehealth clinical encounters.^26^ In addition to randomized studies, integration of wearables with electronic medical records could provide comprehensive capture of health outcomes and new ways to monitor treatment outcomes. For example, wearables could capture patient-reported symptoms and associate them with the electronic medical record, which could facilitate a better understanding of the association of wearables with health outcomes.

### Limitations

This study has several limitations. The study included a single center, and patterns at other centers could differ. Wearable use rates, for example, could be lower in centers that serve poor or more ethnically diverse populations. The characteristics and AF severity among individuals using wearables would be different, in which would be associated with different outcome patterns. Multicenter studies, including randomized studies, are needed to ensure external validity. One cannot infer cause and effect from these exploratory, observational results; using electronic medical record data in this study, we cannot make inferences about the appropriateness of health care use, particularly because we could not reliably ascertain symptoms or why patients sought care.

Exclusions and propensity score matching were used to limit confounding, but residual confounding is a limitation. Because wearables are commercially available, we could not classify patients using orders and instead relied on references to wearable-specific terms in record text. While the group classified as individuals who did not use wearables referred to patients without mention of a wearable, patients may have been misclassified as not using wearables if their clinical notes failed to mention devices. Assuming that use of wearables was associated with increased health care use, misclassification of individuals who used wearables as those who did not might lead to a reduction in the difference in pulse rate and use rate between the 2 groups. In other words, the misclassification could bias the results toward the null. Ultimately, a systematic method to capture individuals who use wearables will be needed. We were unable to ascertain if the specific wearables included rhythm classification software or were limited to pulse measurements. Additionally, composite health care use was defined a priori based on clinical relevance to AF; different definitions could be associated with different results.

## Conclusions

Wearables are an increasing part of health care delivery, particularly in the fields of electrophysiology and cardiology. Many patients with or without AF use wearables for self-directed management or in conjunction with the health care delivery system, so it is important to understand the association of these devices with health outcomes and health care use. This study’s finding suggests that wearable use among patients with AF is associated with increased health care use and support the need for randomized clinical trials to measure the impact of wearables on health outcomes and use among patients with AF.

## Notes

Supplement. **eFigure.** Counts of Device Terms Identified in Patient Clinical Notes **eTable 1.** Search Terms Used to Identify Candidate Patients Using Wearable or Mobile Devices (Case Insensitive) **eTable 2.** Standardized Difference of Covariates Before and After Propensity Matching **eTable 3.** Mixed-effects Linear Regression for Mean Pulse Rate in 1-Unit and 5-Unit Intervals **eTable 4.** Mixed-effects Linear Regression for Composite Health Care Use Outcome **eTable 5.** Mixed-effects Linear Regression for Median Pulse Rate in 1-Unit and 5-Unit Intervals
