# Supplementary material for: Association of Wearable Device Use With Pulse Rate and Health Care Use in Adults With Atrial Fibrillation
Source: JAMA Netw Open. 2021 May 27;4(5):e215821. doi: 10.1001/jamanetworkopen.2021.5821 (PMC8160588; doi:10.1001/jamanetworkopen.2021.5821)
Supplement: Supplement. — eFigure. Counts of Device Terms Identified in Patient Clinical Notes eTable 1. Search Terms Used to Identify Candidate Patients Using Wearable or Mobile Devices (Case Insensitive) eTable 2. Standardized Difference of Covariates Before and After Propensity Matching eTable 3. Mixed-effects Linear Regression for Mean Pulse Rate in 1-Unit and 5-Unit Intervals eTable 4. Mixed-effects Linear Regression for Composite Health Care Use Outcome eTable 5. Mixed-effects Linear Regression for Median Pulse Rate in 1-Unit and 5-Unit Intervals [file jamanetwopen-e215821-s001.pdf]

Supplemental Online Content

Wang L, Nielsen K, Goldberg J, et al. Association of wearable device use with pulse rate and health care use in adults with atrial fibrillation. *JAMA Netw Open.* 2021;4(5):e215821. doi:10.1001/jamanetworkopen.2021.5821

eFigure. Counts of Device Terms Identified in Patient Clinical Notes
eTable 1. Search Terms Used to Identify Candidate Patients Using Wearable or Mobile Devices (Case Insensitive)
eTable 2. Standardized Difference of Covariates Before and After Propensity Matching
eTable 3. Mixed-effects Linear Regression for Mean Pulse Rate in 1-Unit and 5-Unit Intervals
eTable 4. Mixed-effects Linear Regression for Composite Health Care Use Outcome
eTable 5. Mixed-effects Linear Regression for Median Pulse Rate in 1-Unit and 5-Unit Intervals

This supplemental material has been provided by the authors to give readers additional information about their work.

© 2021 Wang L et al. *JAMA Network Open.*

eFigure. Counts of Device Terms Identified in Patient Clinical Notes

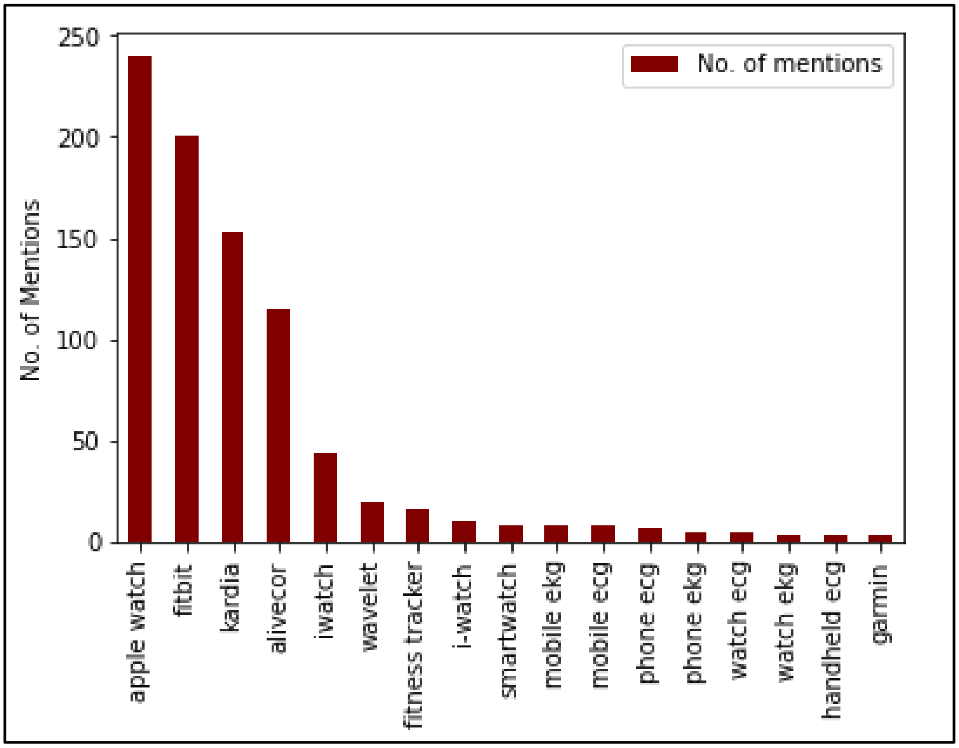

Supplemental Figure Legend. Counts of device terms identified in patient clinical notes. Bars represent the absolute number of mentioned terms in extracted notes from medical records of unique patients. The most commonly mentioned devices were apple, fitbit, kardia, and alivecor.

© 2021 Wang L et al. *JAMA Network Open.*

eTable 1. Search Terms Used to Identify Candidate Patients Using Wearable or Mobile Devices (Case Insensitive)

smartwatch, smart watch, apple-watch, apple watch, applewatch, fitbit, fit-bit, fit bit, heart-rate tracker, wearable, wearable heart monitor, alivecor, kardiaband, kardia, kardia-band, kardia band, watch ecg, watch-ecg, watch ekg, watch-ekg, wrist ecg, wrist ekg, wrist-ecg, wrist-ekg, fitness tracker, iwatch, i-watch, biostrap, wavelet, handheld ecg, handheld ekg, band, mobile ecg, mobile ekg, mobile phone, mobile-phone, smartphone, smart phone, smart-phone, iphone

© 2021 Wang L et al. *JAMA Network Open.*

eTable 2. Standardized Difference of Covariates Before and After Propensity Matching

Matched Covariate	Standardized Difference Prior to Matching	Standardized Difference After Matching
**At least one prior ablation CPT code**	0.601	0.084
**Days since 1st AF diagnosis**	0.292	-0.039
**First AF visit**	-0.898	-0.018
**Charlson comorbidity index <=2**	-0.047	-0.006
**Charlson comorbidity index 3-4**	0.099	0.034
**Charlson comorbidity index >=5**	0.217	-0.009
**No. prior emergency room visits**	0.313	0.055
**CHA_2DS_2-VASc score >1**	-0.011	-0.016
**CHA_2DS_2-VASc score missing**	-0.047	-0.006
**No. cardioversion CPT code**	0.745	0.073
**Age, years**	-0.591	-0.098
**Index Year**	0.387	0.041
**No. prior cardioversion CPT codes**	0.596	0.04
**No. prior E&M CPT codes, mean**	0.382	0.022
**At least one prior rate control drug order**	0.202	-0.042
**At least one prior antiarrhythmic drug order**	0.382	-0.014
**At least one prior telephone note**	0.453	-0.007
**Baseline heart rate >109bpm**	-0.049	0
**Prior OAC treatment (orders and medication list)**	0.536	0.059
**Female Sex**	0.008	0.031
**Area deprivation index**	-0.511	0.016
AF=atrial fibrillation; CPT=Common Procedural Terminology; E&M=evaluation and management; OAC=oral anticoagulant		

© 2021 Wang L et al. *JAMA Network Open.*

eTable 3. Mixed-effects Linear Regression for Mean Pulse Rate in 1-Unit and 5-Unit Intervals

**Model Summary**					
	**No. of Patients**	**Mean**	**Std. Dev.**	**Std. Err.**	**95% CI**
**1-Unit Intervals**					
**Non-User**	500	75.79	12.67	0.57	(74.68, 76.91)
**User**	125	75.01	12.88	1.15	(72.73, 77.29)
**5-Unit Intervals**					
**Non-User**	500	15.16	2.53	0.11	(14.94, 15.38)
**User**	125	15.00	2.58	0.23	(14.55, 15.46)
**Model Estimates**					
	**Coefficient**	**Std. Err.**	**z**	**P>|z|**	**95% CI**
**1-Unit Intervals**					
**Intercept**	75.79	0.57	133.35	0.000	(74.68, 76.91)
**User**	-0.79	1.27	-0.62	0.54	(-3.28, 1.71)
**Group Var**	0.00	0.48			
**5-Unit Intervals**					
**Intercept**	15.16	0.11	133.35	0.000	(14.94, 15.38)
**User**	-0.16	0.25	-0.62	0.54	(-0.66, 0.34)
**Group Var**	0.00	0.10			
Abbreviations: CI, Confidence Interval; Var, Variance					

© 2021 Wang L et al. *JAMA Network Open.*

eTable 4. Mixed-effects Linear Regression for Composite Health Care Use Outcome

**Model Summary**					
	**No. of Patients**	**Mean**	**Std. Dev.**	**Std. Err.**	**95% CI**
**Non-User**	500	3.27	1.42	0.06	(3.14, 3.40)
**User**	125	3.55	1.40	0.13	(3.30, 3.80)
**Model Estimates**					
	**Coefficient**	**Std. Err.**	**z**	**P>|z|**	**95% CI**
**Intercept**	3.27	0.06	51.61	0.000	(3.14, 3.40)
**User**	0.28	0.14	2.01	0.05	(0.01, 0.56)
**Group Var**	0.00	0.05			
Abbreviations: CI, Confidence Interval; Var, Variance					

© 2021 Wang L et al. *JAMA Network Open.*

eTable 5. Mixed-effects Linear Regression for Median Pulse Rate in 1-Unit and 5-Unit Intervals

**Model Summary**					
	**No. of Patients**	**Mean**	**Std. Dev.**	**Std. Err.**	**95% CI**
**1-Unit Intervals**					
**Non-User**	500	75.03	13.32	0.60	(73.86, 76.20)
**User**	125	73.68	12.74	1.14	(71.42, 75.94)
**5-Unit Intervals**					
**Non-User**	500	15.01	2.66	0.12	(14.77, 15.24)
**User**	125	14.74	2.55	0.23	(14.28, 15.19)
**Model Estimates**					
	**Coefficient**	**Std. Err.**	**z**	**P>|z|**	**95% CI**
**1-Unit Intervals**					
**Intercept**	75.03	0.59	127.04	0.000	(73.87, 76.19)
**User**	-1.35	1.32	-1.02	0.31	(-3.94, 1.24)
**Group Var**	0.00	0.60			
**5-Unit Intervals**					
**Intercept**	15.01	0.12	127.04	0.000	(14.78, 15.24)
**User**	-0.27	0.26	-1.02	0.31	(-0.79, 0.25)
**Group Var**	0.00	0.12			
Abbreviations: CI, Confidence Interval; Var, Variance					

© 2021 Wang L et al. *JAMA Network Open.*
